# Frequency and Mortality of Adult Meningitis, Pneumonia, or Bacteremia in Colombia from 2015 to 2022: A Retrospective Database Study in a Health Maintenance Organization

**DOI:** 10.36469/001c.141461

**Published:** 2025-09-17

**Authors:** Juan M. Reyes Sánchez, Carlos Bello, Jhon Bolaños López, Jair Arciniegas, Farley J. González, Manuela Duque, Jose M. Oñate, Mónica García, Omar Escobar, Lidia Serra, Jennifer Onwumeh-Okwundu, Florence Lefebvre d’Hellencourt, Jorge La Rotta, Mark A. Fletcher

**Affiliations:** 1 Pfizer, Bogotá, Colombia; 2 Centro de Biociencias Sura, Medellín, Colombia; 3 Pfizer, Newtown Square, Pennsylvania, USA; 4 Pfizer, Collegeville, Pennsylvania, USA; 5 Pfizer, Paris, France

**Keywords:** bacteremia, pneumonia, comorbidities, burden on disease, meningitis

## Abstract

**Background:**

Meningitis, pneumonia, and bacteremia, prevalent community-acquired diseases that can lead to multi-organ failure, are influenced by age, comorbidities, and living conditions. Despite meningitis surveillance in Colombia, information on pneumonia and bacteremia remains limited. This study aims to determine frequency of these diseases among Colombian patients and estimate related healthcare resources.

**Objective:**

To measure the frequency and mortality of meningitis, pneumonia, and bacteremia in all diagnosed adult patients in Colombia from 2015 to 2022.

**Methods:**

This retrospective study analyzed adult (≥18 years) patients, from structured data collection (International Classification of Diseases, Tenth Revision) in a health maintenance organization (HMO). Diagnosis of a first meningitis, pneumonia, or bacteremia episode—unknown cause (bacterial etiology undetermined)—between 2015 and 2022 was reviewed. Index date was defined as when the diagnosis was registered. Frequency was calculated by dividing the number of cases by the number of members in the HMO system over the study period.

**Results:**

Among 112 205 patients, 96.0% had pneumonia, 6.2% bacteremia, and 0.4% meningitis, not mutually exclusive. Inpatient pneumonia incidence, which peaked in 2019 and dropped post-COVID pandemic, was 167 cases per 100 000 person-years in 2022. Incidence of meningitis, pneumonia, and bacteremia was higher in patients over 60 years. Common comorbidities were chronic obstructive pulmonary disease and cardiovascular disease. Bacteremia incidence decreased from 143 cases per 100 000 in 2015 to 69.6 in 2022. Meningitis incidence dropped from 5.3 to 2.2 cases per 100 000 in the COVID period. All-cause mortality rates were 12.0%, 33.5% and 13.8% for pneumonia, bacteremia, and meningitis, respectively.

**Discussion:**

This study is the first to use health electronic databases from an HMO to estimate the burden of these diseases in Colombian patients. Incidence was consistent with COVID-period patterns observed in other studies. Mortality rates were higher with bacteremia. Comorbidities like chronic pulmonary disease, cardiovascular disease, kidney diseases, and dementia were linked with increased incidence and mortality, emphasizing the need for targeted healthcare interventions and vaccination programs.

**Conclusion:**

Incidence and mortality, whether pneumonia (inpatient or outpatient), bacteremia, or meningitis with bacteremia, vary with age and comorbidities, while all-cause mortality was greater for bacteremia than pneumonia or meningitis.

## BACKGROUND

Meningitis, pneumonia, and bacteremia are diseases acquired in community settings that are prevalent worldwide and represent a substantial public health concern due to potential complications like multiorgan failure.^1,2^

The age-standardized incidence of meningitis in populations with low sociodemographic status was estimated at 82.3 cases per 100 000 persons per year in 2019 with a mortality rate of 11.2 deaths per 100 000 persons per year. In the Andean region of Latin America, which includes Colombia, the incidence estimate in 2019 was 6.9 cases per 100 000 persons per year and a mortality rate of 0.6 deaths per 100 000 persons per year.^3^ The main etiologies of meningitis were viruses, *Neisseria meningitides*, and *Streptococcus pneumoniae.*^3^ For 2022, there were 1116 notified cases of bacterial meningitis, which represented an increase of 58.5% over the notifications made in 2021; however, it could be influenced by the post-COVID pandemic situation.^4^

The incidence of inpatient pneumonia has been reported in developing countries as 4.9 episodes per 1000 persons per year in patients aged 65 to 74 years, 14.6 episodes per 1000 persons per year in patients aged 75 to 84, and 45.6 episodes per 1000 persons per year in those older than 85 years. The case-fatality rate observed was 9.0% for pneumonia hospitalization patients aged 65 to 74 years, 12.1% in those 75 to 84 years, and 17.5% for those aged 85 years and older.^5^

Comorbidities are the main risk factors for meningitis, along with environmental and social variables. Close contact or overcrowding settings, such as attending university, attending daycare, or living in densely populated areas, raises possible exposure to the pathogen and hence increases the risk of infection. Older adults have been identified as a population at higher risk for acquiring meningitis due to their debilitated immune system and higher rates of comorbidities; additionally, vaccine efficacy tends to be lower in this age group.^6,7^

Furthermore, alterations in the immune system, such as those caused by immunosuppressive medications and HIV infection, can increase relative risk up to a hundred-fold. Despite the availability of antiviral drugs, patients living with HIV remain at higher risk than the general population. Patients with hematologic malignancies or those who have recently had neurosurgery also are at higher risk of contracting meningitis.^8,9^ Moreover, immunosuppressive therapies have been recognized as risk factors for invasive bacterial infections.^6^

Pneumonia has similar risk factors to meningitis such as advanced age, smoking, and immunosuppressive conditions. Other risk factors for this disease are alcoholism or underlying conditions like chronic obstructive pulmonary disease, cerebrovascular disease, cardiovascular disease, diabetes mellitus, dementia, and chronic liver or renal disease. A literature review also identified being underweight with an increased risk of pneumonia relative to normal body weight.^10,11^ Similar to meningitis, overcrowding, especially in the household, was also associated with increased risk.^10^

The incidence of bacteremia reported in United States and Canada is 113 to 189 cases per 100 000 persons per year, while in European countries it can vary between 166 and 220 cases per 100 000 persons per year.^12^ The most common bacteria that cause healthcare-associated bacteremia are *Staphylococcus aureus, Escherichia coli, Klebsiella* spp, and *Pseudomonas aeruginosa,* while in community-acquired cases the most common bacteria are *S. pneumoniae,* other streptococci, and *E. coli.*^12^ Risk factors for bacteremia include having a weakened immune system, undergoing certain medical procedures, and having an infection in another part of the body.^13,14^

Currently, there is a gap in information in Colombia about the distribution and frequency of meningitis bacteremia and pneumonia and their association with risk factors. Although there is a national policy of meningitis surveillance by governmental agencies, this study aims to determine the frequency of these infections in one of largest health maintenance organizations (HMOs) in Colombia and to estimate the use of resources to treat these infections and any resulting diseases.

Data from HMOs in Colombia represent a valuable source of information. These organizations act as intermediaries between patients and the Colombian government; consequently, they maintain large and well-organized information systems with reliable and continuously updated databases.

## METHODS

### Study Design and Population

A retrospective, descriptive study on patients of age 18 years or more diagnosed with meningitis, pneumonia, or bacteremia who received both inpatient and outpatient medical care in one HMO in Colombia was conducted. The HMO is the third largest health insurer in Colombia, covering 30 of the 33 departments of the country and more than 5 million people (~10% of the population).

Patient inclusion criteria were age older than 18 years and a first coding registered, based on the *International Classification of Diseases, Tenth Revision* [ICD 10], of pneumonia (J13, J14, 15.6, J15.8, J15.9, J16.8, J18.0, J18.1, J18.9, J22), bacteremia (A39.4, A39.9, A40.3, A41.3, A41.5, A41.8, A41.9, A49.2, A49.8, A49.9, B95.3), and meningitis (A39.0, G00.0, G00.1, G00.2, G00.9, G03.9) between 2015 and 2022. These definitions for the infectious disease under consideration, although not specific to an etiology, are consistent with definitions used in previous research.^15^ Rehospitalizations for these conditions are not included in this study.

All members of the HMO (ie, the people insured by the HMO) were included in the study from 2015 to 2022. The index date was defined as the date of the first diagnosis of any infection of interest. All patients were followed until end of follow-up, death, or end of observation period on July 31, 2023. The follow-up period was considered to be the index date to the end of follow-up, depending on the case. In the outpatient cases, the follow-up was during the acute phase or 60 days after the index date. For inpatient cases, both hospitalization course until discharge and outpatient care in the 60 days after index date were followed. Hospitalization was defined as a length of a hospital stay of at least 24 hours.

The study was presented and approved by the ethics committee of the HMO in Colombia. Since the research was of minimal risk and without intervention in the patient, informed consent was not required.

### Data Sources and Variables

Data was obtained from the HMO databases, which mainly involves medical charts, claims database, laboratories, and diagnostic images. Patients were not contacted. The data available were diagnoses, age, sex, geographic region of residence, hospitalization if required, among others. The HMO’s data management system joins different related databases, which are accessed through Teradata storage space.

Comorbidities were selected based on pneumococcal vaccine recommendations of the 2023 Advisory Committee on Immunization Practice, which differentiate by patient risk group.^16^ It included chronic heart disease, chronic liver disease, chronic lung disease, chronic renal failure, asplenia, leukemia, lymphoma malignancy, among others.

### Outcomes

Pneumonia, bacteremia, and meningitis episodes were defined as one or more outpatient and/or inpatient claims with an ICD-10 diagnosis with unknown cause (bacterial etiology not determined).

Case fatality rates (CFRs) were defined as the proportion of deaths among disease episodes. It was calculated by number of deaths in each period, divided by the number of cases found for the specific disease in the same study period. Mortality was defined as the proportion of deaths among the entire population in the year of study. It was calculated as number of deaths divided by all members of the HMO considered to be at risk in each year.

### Statistical Analysis

The statistical analysis focused on descriptive statistics, where qualitative variables were presented as frequencies and percentages, while continuous quantitative variables were summarized using mean and SD. Negative binomial regression was used to determine the variability of incidence, mortality, and CFR values across age groups and comorbidities. This method was selected based on the nature of the aggregated count data available for the analysis and the presence of overdispersion. The *P* values resulted from likelihood ratio tests (type II ANOVA with chi-square test) of the regression were reported to assess whether age groups and comorbidities were significantly associated with changes or differences in incidence. Details on the model can be found in the **Online Supplemental Material**.

By groups of age, the differences in incidence between comorbidities were analyzed using a 2-proportion *z*-test. Level of significance was adjusted with Bonferroni correction. Additionally, a 2-proportion *z*-test was used for testing statistical differences in incidences by sex rather than the negative binomial regression model because of limitations in data disaggregation.

Annual cumulative incidence, mortality, and CFR were calculated from the number of events (pneumonia, bacteremia, and meningitis or deaths caused by these diseases) in the specific period. The annual incidence rate was calculated as [(New Cases/Population) × 100 000] based on the number of patients with events divided by all members of HMO at risk for each year. The population was defined as all active members in the HMO during the year of estimation. The age-standardized values were calculated using the direct method from age-specific rates in the study population, which was then used to calculate the weighted average for the total population.^17^ The significance level was set at 5%. Statistical analysis was performed using R (v. 4.3.1) using different packages (dplyr, data.table, epiR, janitor, MASS, and nlme).^18^

## RESULTS

During the study period, 112 205 cases were identified. The distribution of cases by proportion was 0.4% for meningitis (412), 96.0% for pneumonia (107 675), and 6.2% for bacteremia (7006). **Table 1** summarizes the clinical and demographic characteristics of the identified patients, categorized by diagnosis and disease status.

**Table 1.** Clinical and Demographic Characteristics of Included Patients

**Variables**	**Pneumonia**		**Meningitis**	**Bacteremia**
	**Hospital**	**Ambulatory**		
No. of cases	15 196	92 479	412	7006
Age, mean (SD)	63.1 (19.7)	51.4 (19.5)	45.1 (18.7)	60.9 (20.2)
Age group, n (%)				
18-49 y	3739 (24.6)	438598 (47.1)	258 (62.6)	2001 (28.6)
50-59 y	2167 (14.3)	14541 (15.7)	52 (12.6)	1013 (14.5)
60-69 y	2686 (17.7)	16261 (17.6)	46 (11.2)	1227 (17.5)
70-79 y	2997 (19.7)	9807 (10.6)	37 (9.0)	1347 (19.2)
>80 y	3607 (23.7)	8271 (8.9)	19 (4.61)	1418 (20.2)
Female gender, n (%)	7981 (52.5)	54278 (58.7)	190 (46.1)	3830 (54.7)
ICU, n (%)	1817 (12.0)		14 (3.4)	485 (6.92)
Comorbidities, n (%)				
None	1811 (11.9)	27909 (30.2)	133 (32.3)	1037 (14.8)
Dementia	2252 (14.8)	4996 (5.4)	40 (9.7)	1018 (14.5)
Hypertension	10 299 (67.8)	43 414 (46.9)	161 (32.3)	4708 (67.2)
Myocardial infarction	1286 (8.5)	3684 (4.0)	16 (3.9)	611 (8.7)
Peripheral vascular disease	836 (5.5)	2883 (3.1)	13 (3.2)	429 (6.1)
Congestive heart failure	416 (2.7)	1197 (1.3)	5 (1.2)	172 (2.5)
Stroke or cerebrovascular disease	359 (2.4)	942 (1.0)	14 (3.4)	168 (2.4)
Connective tissue disease	1299 (8.5)	5906 (5.6)	35 (8.5)	608 (8.7)
Chronic obstructive pulmonary disease	8112 (53.4)	26 994 (29.2)	64 (15.5)	2544 (36.6)
Asthma	2532 (16.7)	14 013 (15.2)	27 (6.6)	525 (7.5)
Emphysema	180 (1.2)	552 (0.6)	1 (0.2)	39 (0.6)
Diabetes	4680 (30.8)	17 817 (19.3)	61 (14.8)	2345 (33.5)
Diabetes with organ damage	1505 (9.9)	4243 (4.6)	19 (4.6)	937 (13.4)
Hepatic disease, any stage	498 (3.3)	3212 (3.5)	13 (3.2)	352 (5.0)
Hepatic disease, moderate/severe	34 (0.2)	129 (0.1)	0 (0.0)	30 (0.4)
Asplenia	4 (0.0)	8 (0.0)	0 (0.0)	2 (0.0)
HIV infection	422 (2.8)	2998 (3.2)	39 (9.5)	197 (2.8)
Solid tumor	2903 (19.1)	12 961 (14)	71 (17.2)	1577 (22.1)
Leukemia	323 (2.1)	554 (0.6)	9 (2.2)	186 (2.7)
Malignant lymphoma	244 (1.6)	701 (0.8)	7 (1.7)	137 (2.0)
Chronic kidney disease (any stage)	3495 (23)	9603 (10.4)	50 (12.1)	2155 (30.8)
Organ transplant	93 (0.6)	264 (0.3)	3 (0.7)	58 (0.8)
Hemiplegia or paraplegia	240 (1.6)	577 (0.6)	16 (3.9)	212 (3.0)
Immunosuppressive treatment	1009 (6.6)	4206 (4.5)	26 (6.3)	471 (6.7)
No. of comorbidities, n (%)				
0	1811 (11.9)	27 909 (30.2)	133 (32.3)	1037 (14.8)
1	2116 (13.9)	20 707 (22.4)	97 (23.5)	925 (13.2)
2	2565 (16.9)	15 503 (16.8)	62 (15.0)	1089 (15.5)
3	2636 (17.3)	11 542 (12.5)	45 (10.9)	1127 (16.1)
>3	6068 (39.9)	16 818 (18.2)	75 (18.2)	2828 (40.4)

Abbreviation: ICU, intensive care unit.

### Meningitis

The incidence of meningitis during the pre-COVID period was estimated to be between 3.5 to 5.3 cases per 100 000 person-years and 2.2 to 3.7 cases per 100 000 person-years in the COVID period. CFR was 4.2% to 13.3% in all age groups, and the mortality rate was 0.1 to 0.3 deaths per 100 000 person-years with mild variation between years. The incidence increased from 2.8 cases in the 18- to 49-year age range to 11.9 cases per 100 000 person-years in patients older than 70 years. Similarly, the CFR and mortality increased with age; however, there was no statistical difference in mortality or incidence (**Table 2**).

**Table 2.** Incidence, Case Fatality Rate, and Meningitis Mortality

**Variable**	**Incidence^a^**	**CFR (%)**	**Mortality^b^**	***P* Value**
Year of diagnosis				
Pre-COVID				
2015	5.3 (4.3-6.5)	6.2 (2.3-13.5)	0.3 (0.1-0.7)	
2016	3.5 (2.7-4.4)	4.2 (0.9-12.3)	0.1 (0.0-0.4)	
2017	4.4 (3.6-5.4)	6.9 (2.8-14.3)	0.3 (0.1-0.6)	
2018	3.9 (3.1-4.7)	6.1 (2.3-13.3)	0.2 (0.1-0.5)	
2019	4.1 (3.4-5.0)	5.7 (2.3 -11.8)	0.2 (0.1-0.5)	
COVID				
2020	2.2 (1.7-2.7)	13.3 (6.4-24.5)	0.3 (0.1-0.5)	
2021	2.8 (2.3-3.4)	7.5 (3.2-14.7)	0.2 (0.1-0.4)	
2022	3.7 (3.1-4.3)	8.6 (4.6-14.6)	0.3 (0.2-0.5)	
Age range				
18-49 y	2.8 (2.5-3.1)	4.8 (2.9-7.7)	0.1 (0.1-0.2)	
50-59 y	3.0 (2.4-3.6)	8.1 (3.4-17.0)	0.3 (0.1-0.6)	Incidence: .751 CFR: NA Mortality: .146
60-69 y	4.7 (3.8-5.7)	6.6 (2.8-14.1)	0.4 (0.2-1.0)	
≥70 y	11.9 (10.2-13.7)	12.4 (7.9-18.8)	1.6 (9.9-23.9)	
Sex				
Female	1.6 (1.3-1.8)	12.1 (0.9-16.5)	0.2 (0.2-0.3)	Incidence: .003 CFR: .743 Mortality: .144
Male	2.1 (1.9-2.5)	21.0 (16.1-27.4)	0.3 (0.2-0.4)	
Comorbidities (2015-2022)				
Dementia	43.6 (31.1-59.3)	35 (19.1-58.7)	15.2 (8.3-25.6)	
Cardiovascular disease	47.4 (33.1-66.0)	11.4 (3.1-29.3)	5.4 (1.5-1.4)	
Chronic pulmonary disease	19.4 (15.7-23.8)	30.4 (20.2-44.0)	5.9 (3.9-8.6)	
Connective tissue disease	27.9 (19.4-38.8)	11.4 (3.1-29.3)	3.2 (0.9-8.2)	
Peripheral vascular disease	49.4 (26.3-84.5)	23.1 (4.4-67.4)	11.4 (2.4-33.3)	
Diabetes	12.0 (9.5-14.9)	36.2 (24.3-52.1)	4.3 (2.9-6.2)	Incidence: NA CFR: NA Mortality: NA
Hepatic disease	19.4 (10.3-33.1)	23.1 (NA-NA)	4.5 (0.9-13.1)	
Asplenia	0.0 (0.0-0.0)	NA	NA	
HIV infection	92.4 (65.7-126.0)	20.5 (NA-NA)	6.5 (0.2-36.3)	
Cancer	23.1 (18.5-28.4)	25.3 (15.8-38.3)	5.8 (3.7-8.8)	
Kidney disease any stage	60.0 (44.5-79.0)	32 (18.3-52)	19.2 (11-31.2)	
Organ transplant	19.4 (4.0-57.1)	33.3 (NA-NA)	6.52 (0.2-36.3)	
Hemiplegia or paraplegia	331.0 (189.0-537.0)	12.5 (1.5-45.2)	41.3 (5.0-149)	

Abbreviations: CFR, case fatality rate; NA, not available.

^a^Cases per 100 000 person-years.

^b^Deaths per 100 000 person-years.

**Figure 1.**
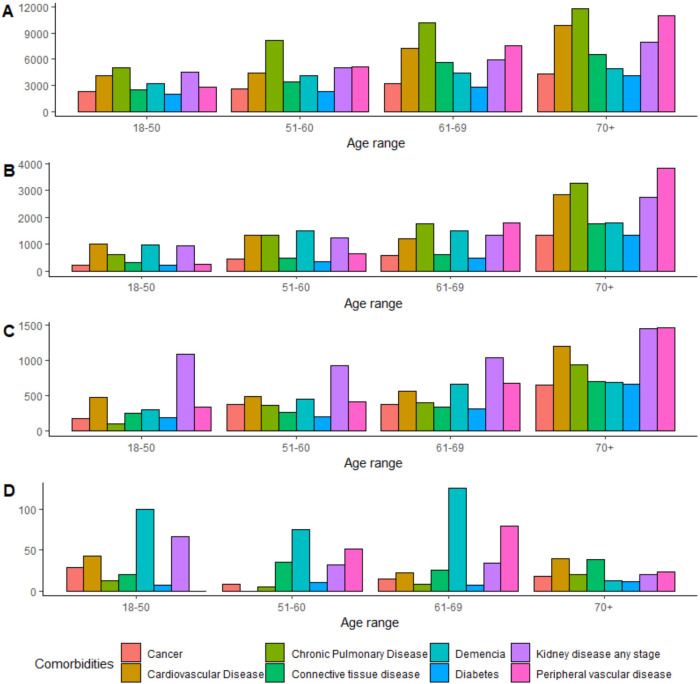
Incidence of Ambulatory Pneumonia (**A**), Hospital Pneumonia (**B**), Bacteremia (**C**), and Meningitis (**D**)

The main comorbidities with an incidence of more than 40 cases per 100 000 person-years were cardiovascular diseases, dementia, kidney disease, peripheral vascular disease, HIV, and hemiplegia-paraplegia. It was not possible to estimate the incidence of asplenia. Within the CFR, the frequency of chronic pulmonary disease, dementia, diabetes, kidney disease, and transplant was more than 30% while dementia, kidney disease, peripheral vascular diseases, and hemiplegia-paraplegia had mortality rates of over 10 deaths per 100 000 person-years (**Table 2**). Dementia had the highest incidence in patients younger than 70 years, even in patients between 18 to 50 years, and peripheral vascular disease was highest in patients between 50 to 70 years of age. However, the incidence in patients older than 70 years was not higher compared with other age categories (**Figure 1**).

Pairwise comparison tests by comorbidity in meningitis disease were not presented due to the observed low incidence, which reduces the power of the *z*-test being unable to detect small differences. Nonetheless, numerical differences could be observed: “hemiplegia or paraplegia” and HIV had the first and second-highest incidences, respectively, with a relative difference as compared to other comorbidities like dementia, cardiovascular disease and peripheral vascular disease, and diabetes had the lowest incidence.

### Pneumonia

About one-seventh of all pneumonia cases resulted in hospitalization; The mean (SD) age of inpatient pneumonia patients was 63.1 (19.7) years. Patients who were aged 18 to 49 years old, over 80 years, or female were the most frequently represented groups. Twelve percent of inpatient pneumonia cases required intensive care unit care. In contrast, outpatient pneumonia cases involved younger population, with a mean age of 51.4 (19.5) years. The most represented groups among outpatients were females and patients aged 18 to 49 years.

The incidence of inpatient pneumonia was higher during the pre-COVID period (2015-2019) compared with the post-COVID period (2020-2022). However, the latter period showed a recovery trend every year. The CFR was higher in the post-COVID period (**Table 3**). The incidence, CFR, and mortality increased with patient age, with significant differences between age groups (*P* < .001). Inpatient pneumonia incidence, but not CFR and mortality, depended on patient gender (*P*=.009, .829, and .204, respectively). Asplenia, kidney disease, peripheral vascular disease, and hemiplegia/paraplegia were associated with incidence rates of inpatient pneumonia greater than 3000 cases per 100 000 person-years, while cardiovascular disease, chronic pulmonary disease, and dementia were over 2000 cases per 100 000 person-years. Underlying comorbidities with CFRs over 30% were cancer, cardiovascular diseases, chronic pulmonary disease, dementia, diabetes, kidney disease, peripheral vascular disease, hemiplegia-paraplegia, and transplantation (**Table 3**).

**Table 3.** Incidence, Case Fatality Rate, and Inpatient Pneumonia Mortality

**Variable**	**Incidencea**	**CFR (%)**	**Mortalityb**	***P* Value**
Year of diagnosis				
Pre-COVID				
2015	270 (262-278)	10.1 (9.2-11)	24.2 (24.8-29.7)	
2016	235 (228-242)	9.1 (8.3-10)	21.4 (19.4-23.5)	
2017	257 (251-264)	9.8 (9.0-10.6)	25.1 (23.1-27.3)	
2018	243 (237-249)	9.9 (9.1-10.7)	23.9 (22.1-25.9)	
2019	240 (235-246)	12.2 (11.4-13.0)	29.3 (27.4-31.4)	
COVID				
2020	134 (130-138)	13.5 (12.4-14.6)	18.0 (16.6-19.5)	
2021	120 (117-124)	12.2 (11.2-13.2)	14.6 (13.4-15.9)	
2022	167 (163-171)	9.9 (9.2-10.7)	16.6 (15.4-17.9)	
Age range				
18-49 y	50.8 (49.6-52.1)	4.8 (4.3-5.4)	2.3 (2.0-2.6)	
50-59 y	195 (190-200)	6.6 (6.0-7.3)	12.9 (11.6-14.3)	Incidence: <.001 CFR: <.001 Mortality: <.001
60-69 y	321 (314-329)	8.5 (7.8-9.2)	26.3 (24.2-28.5)	
≥70 y	1390 (1370-1410)	14.5 (14.0-15.0)	198 (191-205)	
Sex				
Female	685 (669-701)	32.1 (30.8-33.4)	22.1 (21.2-23.0)	Incidence: .009 CFR: .829 Mortality: .204
Male	691 (674-707)	31.8 (30.5-33.2)	22.0 (21.1-22.9)	
Comorbidities (2015-2022)				
Dementia	2450 (2350-2560)	64.7 (61.5-68.2)	1590 (1510-1670)	
Cardiovascular disease	2790 (2670-2920)	47.3 (44.3-50.3)	1320 (1240 -1410)	
Chronic pulmonary disease	2290 (2240-2330)	34.4 (33.3-35.5)	786 (761-812)	
Connective tissue disease	1040 (980-1090)	28.5 (25.7-31.5)	295 (266-327)	
Peripheral vascular disease	3180 (2970-3400)	40.1 (35.9-44.6)	1270 (1140-1420)	
Diabetes	927 (904-951)	38.7 (37.2-40.3)	359 (345-374)	Incidence: .001 CFR: <.001 Mortality: <.001
Hepatic disease	792 (726-863)	28.8 (24.4-33.7)	228 (193-267)	
Asplenia	4400 (1200-11300)	0	1100 (27.8-612)	
HIV infection	999 (906-1100)	18.2 (14.4-22.8)	182 (144-228)	
Cancer	920 (889-951)	45.1 (42.9-47.4)	415 (395-436)	
Kidney disease any stage	4190 (4050-4330)	43.2 (41.1-45.4)	1810 (1720-1900)	
Organ transplant	606 (489-742)	32.3 (21.8-46.1)	195 (132-279)	
Hemiplegia or paraplegia	4960 (4350-5630)	37.1 (29.8-45.6)	1840 (1480-2260)	

Abbreviations: CFR, case fatality rate; NA, not available.

^a^Cases per 100 000 person-years.

^b^Deaths per 100 000 person-years.

Unfortunately, the changes in incidence related to the number of comorbidities were not possible to analyze; however, it was observed that inpatient pneumonia increased with the number of comorbidities (**Table 1**). Incidence and mortality of pneumonia per age and comorbidities are presented in **Supplementary Tables S1-S4.** For people aged 51 to 69, only a few comorbidities (eg, chronic pulmonary disease, cardiovascular diseases, and dementia) significantly influenced inpatient pneumonia incidence. In contrast, for those aged 70 and older, nearly all comorbidities had a substantial effect (**Table S2**).

Incidence for outpatient pneumonia ranged from 747 to 1030 cases per 100 000 person-years during the pre-COVID period, which was higher than the post-COVID period. CFR and mortality were similar in both periods with a slight decrease in 2022. CFR increased with age and was higher in women than men, while mortality rate was influenced by age but not by sex. There was a statistically significant difference in the incidence and CFR of the disease in at least one of the comorbidities (*P* < .001). Kidney disease, peripheral vascular disease, and hemiplegia-paraplegia were found with highest incidence of outpatient pneumonia, follow up by splenia, cardiovascular disease, chronic pulmonary disease, and HIV (**Table 4**). The incidence of hronic pulmonary disease differed from all other comorbidities (**Table S3**). For both inpatient and outpatient pneumonia, chronic pulmonary diseases, cardiovascular diseases, kidney disease, and peripheral vascular disease were more prevalent in patients older than 60 years compared with other comorbidities (**Figure 1**).

**Table 4.** Incidence, Case Fatality Rate, and Outpatient Pneumonia Mortality

**Variable**	**Incidence^a^**	**CFR (%)**	**Mortality^b^**	***P* Value**
Year of diagnosis				
Pre-COVID				
2015	747 (735-760)	4.5 (4.2 -4.9)	33.7 (31.1-36.5)	
2016	788 (776-801)	4.0 (3.7-4.3)	31.2 (28.8-33.7)	
2017	986 (973-999)	3.8 (3.6-4.1)	37.7 (35.2-40.3)	
2018	1010 (1000-1020)	4.1 (3.9-4.4)	41.7 (39.3-44.3)	
2019	1030 (1020-1040)	4.9 (4.6-5.1)	50 (47.5-52.6)	
COVID				
2020	637 (629-646)	4.8 (4.5-5.1)	30.4 (28.6-32.3)	
2021	864 (855-874)	5.3 (5.0-5.5)	45.6 (43.4-47.8)	
2022	652 (645-660)	3.9 (3.6-4.1)	25.2 (23.7-26.8)	
Age range				
18-49 y	404 (401-408)	1.0 (0.9-1.1)	4.0 (3.6-4.3)	
50-59 y	954 (944-965)	2.3 (2.1-2.5)	21.6 (20.0-23.4)	Incidence: <.001 CFR: <.001 Mortality: <.001
60-69 y	1470 (1450-1480)	3.3 (3.1-3.5)	49.1 (46.3-52.0)	
≥70 y	3360 (3330-3380)	9.7 (9.4-9.9)	328 (320-337)	
Sex				
Female	445 (441-448)	9.3 (9.1-9.5)	37 (36.2-37.8)	Incidence: <.001 CFR: <.001 Mortality: .3387
Male	354 (350-358)	8.7 (8.5-8.9)	36.9 (36.1-37.8)	
Comorbidities (2015-2022)				
Dementia	5440 (5290-5590)	47.3 (45.4-49.3)	2570 (2470-2680)	
Cardiovascular disease	7890 (7690-8100)	28.4 (27.1-29.8)	2240 (2140-2350)	
Chronic pulmonary disease	8780 (8690-8860)	15.5 (15.1-15.8)	1360 (1320-1390)	
Connective tissue disease	4710 (4590-4830)	9.7 (8.9-10.5)	455 (419-494)	
Peripheral vascular disease	11 000 (10 600-11 400)	20.5 (18.9-22.2)	2250 (2070-2440)	
Diabetes	3310 (3260-3350)	17.0 (16.5-17.6)	564 (546-582)	Incidence: <.001 CFR: <.001 Mortality: <.503
Hepatic disease	4980 (4810-5150)	8.1 (7.2-9.2)	405 (358-456)	
Asplenia	8790 (380-17300)	0	1100 (27.8-6120)	
HIV infection	7100 (6850-7360)	4.8 (4.1-5.7)	341 (288-401)	
Cancer	3770 (3710-3830)	22.3 (21.5-23.1)	840 (811-870)	
Kidney disease any stage	11 500 (11 300-11 700)	23.6 (22.7-24.6)	2720 (2610-2840)	
Organ transplant	1720 (1520-1940)	19.7 (14.7-25.8)	339 (253-444)	
Hemiplegia or paraplegia	11 900 (11 000-12 900)	20.1 (16.6-24.1)	2400 (1980-2870)	

Abbreviations: CFR, case fatality rate; NA, not available.

^a^Cases per 100 000 person-years.

^b^Deaths per 100 000 person-years.

### Bacteremia

The incidence of bacteremia was 95.9 to 143 cases per 100 000 person-years pre-COVID and 59.2 to 69.6 cases per 100 000 person-years during the COVID period. Contrary to incidence, the CFR was between 9.9% to 14.0% with an increase after 2019, but the mortality decreased after 2020 from 14.3 deaths per 100 000 person-years to 8.3 to 9.6 deaths per 100 000 person-years during the COVID period. Incidence and CFR were affected by age; however, while incidence was not influenced by sex rather by comorbidities, CFR was affected by sex but not by comorbidity. Mortality range was affected by age and comorbidities, but the difference in sex was not statistically significant. Cardiovascular disease, dementia, kidney disease, peripheral vascular disease, and hemiplegia/paraplegia showed incidence values over 1000 cases per 100 000 person-years and mortality over 500 deaths per 100 000 person-years.

These findings are comparable to an econometric study in Bogota, Colombia, evaluating trends of mortality due to pneumonia from 2004 to 2014, which found an overall mortality rate of 137 per 100 000 population.^19^ Dementia, with a 66.4% prevalence, predominates in CFR compared with the other comorbidities (**Table 5**). As noted with inpatient pneumonia, in over 70% of cases, the patients had multiple comorbidities. Kidney disease, with the highest incidence among all age groups, was the most relevant comorbidity, while cardiovascular disease was more predominant patients older than 60 years (**Figure 1**).

**Table 5.** Incidence, Case Fatality Rate, and Mortality of Bacteremia

**Variable**	**Incidence^a^**	**CFR (%)**	**Mortality^b^**	***P* Value**
Year of diagnosis				
Pre-COVID				
2015	143 (137-148)	10.4 (9.2-11.7)	14.8 (13.1-16.7)	
2016	113 (108-118)	10.6 (9.3-12.0)	11.9 (10.5-13.5)	
2017	111 (107-116)	10.3 (9.1-11.6)	11.4 (10.1-12.9)	
2018	95.9 (92.1-99.8)	9.9 (8.7-11.3)	9.5 (8.4-10.8)	
2019	108 (104-111)	13.2 (12.0-14.6)	14.3 (12.9-15.7)	
COVID				
2020	68.8 (66.1-71.6)	13.9 (12.4-15.5)	9.6 (8.6-10.7)	
2021	59.2 (56.8-61.7)	14.0 (12.5-15.7)	8.3 (7.4-9.3)	
2022	69.6 (67.1-72.2)	12.2 (10.9-13.5)	8.5 (7.6-9.4)	
Age range				
18-49 y	28.4 (27.5-29.4)	5.5 (4.5-6.3)	1.5 (1.3 -1.7)	
50-59 y	94.6 (91.1-98.2)	9.2 (8.2-11.9)	8.6 (7.6-9.8)	Incidence: <.001 CFR: <.001 Mortality: <.001
60-69 y	148 (143-153)	10.8 (9.7-11.9)	15.5 (13.9-17.2)	
≥70 y	590 (578-602)	15.4 (14.6-16.2)	88.4 (84.0-93.0)	
Sex				
Female	32.9 (31.9-34.0)	34.8 (33.4-36.3)	10.8 (10.4-11.3)	Incidence: .069 CFR: 0.001 Mortality: 0.096
Male	31.1 (29.9-32.2)	22.4 (21.5-23.4)	11.3 (10.8-11.8)	
Comorbidities (2015-2022)				
Dementia	1110 (1040-1180)	66.4 (61.5-71.6)	736 (682-794)	
Cardiovascular disease	1290 (1210-1370)	48.8 (44.5 -53.4)	629 (573-689)	
Chronic pulmonary disease	657 (634-680)	45.8 (43.5-48.3)	301 (285-317)	
Connective tissue disease	485 (447-525)	31.9 (27.6-36.7)	155 (134-178)	
Peripheral vascular disease	1360 (1480-1790)	44.1 (38-50.8)	718 (620-828)	
Diabetes	492 (475-509)	41.7 (39.5-43.9)	205 (194-216)	Incidence: <.001 CFR: <.063 Mortality: <.001
Hepatic disease	569 (513-629)	34.3 (28.7-40.7)	195 (163-232)	
Asplenia	2200 (2660-7940)	0	0 (NA-4100)	
HIV infection	466 (404-536)	23.9 (17.5-31.7)	111 (81.8-148)	
Cancer	504 (481-527)	46.3 (43.3-49.5)	233 (218-249)	
Kidney disease any stage	2580 (2480-2700)	43.3 (40.6-46.2)	1120 (1050-1190)	
Organ transplant	378 (287-489)	29.3 (17.1-46.5)	111 (64.5-177)	
Hemiplegia or paraplegia	4380 (3810-5010)	29.2 (22.4-37.5)	1280 (982-1640)	

Abbreviations: CFR, case fatality rate; NA, not available.

^a^Cases per 100 000 person-years.

^b^Deaths per 100 000 person-years.

For the age range 51 to 69 years, almost all comorbidities displayed a similar pattern except for diabetes and kidney disease, which had lowest incidences within this age range (**Table S5**). In patients aged 70 or older (**Table S6**), the comorbidities have a higher impact on the incidence of bacteremia, with similarities between chronic pulmonary disease, cardiovascular disease, kidney disease and peripheral vascular disease. These are statistically different from others, having higher levels.

Incidence and mortality of bacteremia per age and comorbidities are presented in the **Online Supplementary Material**.

## DISCUSSION

### Incidence

This study estimated incidence, CFR, and mortality of pneumonia, bacteremia, and meningitis from 2015 to 2022, which included the pandemic period, in one leading HMO in Colombia. It is the first study that involved the use of health electronic databases from an HMO to estimate the burden of the diseases in Colombian patients.

All 3 parameters of inpatient pneumonia described in this study have been observed in previously published studies from Colombia. A retrospective analysis, conducted in 6 countries, including Colombia, used national databases in 2009 to report incidence in adults with age ranges of 50-64, 65-74, 75-84, and ≥85 years that was 102.2, 325.3, 1028.5, and 4967.5 cases per 100 000 person-years, respectively, and a CFR range from 6.9% to 13.6%, depending on age.^20^ Another study conducted in Colombia measured the mortality rates between 1998 and 2015, using the information from the Colombian National Agency of Statistics, and found an age-standardized mortality rate of between 24 to 29 deaths per 100 000 population, which was higher in men than women. Mortality rates in the different age ranges, as for sex, have the same tendency as reported in this study.^21^

Contrary to inpatient pneumonia, outpatient pneumonia observed in this study was higher than from previous Colombian studies. Buzzo et al reported an incidence for Colombia of between 203 and 2085 cases per 100 000 person-years in the studied age groups, in contrast to 404 to 3360 cases per 100 000 person-years reported in our study.^20^ In Germany, the incidence of outpatient pneumonia in 2015 ranged between 466 and 1053 cases per 100 000 person-years.^22^ The German study was limited to 2015, while the Colombian study contained information after 2015 including the post-COVID period. Also, characteristics of the healthcare system and demographic and clinical characteristics of people affects the difference of incidence between countries. For example, the influenza but not the pneumococcal vaccine is supported by the Colombian Minister of Health,^23^ whereas the German government includes both vaccines.^24^

The results for bacteremia are consistent with a World Health Organization (WHO) report related to burden of diseases of sepsis before 2020, which found an incidence of 189 cases 100 000 person-years, with 124 cases per 100 000 person-years and a CFR of 30.1% for the American region.^25^ One 2015 study in Brazil reported a sepsis incidence of 45.6 cases per 100 000 person-years, a mortality rate of 23.3 deaths per 100 000 person-years, and a CFR of 51.1%. The same study reported that the incidence in patients 18 to 64 years of age was 23.3 cases per 100 000 person-years and 217 cases per 100 000 person-years for those 65 to 84 years of age.^26^

Although the trend and incidence reported in our study is consistent with the WHO report, a Brazilian study found a lower incidence. Several reasons could account for this difference, such as the time frame in which they occurred (2006-2015). Additionally, the etiologies and frequency of bacteremia in different countries could vary, considering that the main causes include *Neisseria meningitis*, *Streptococcus aureus*, *Streptococcus pneumoniae*, and others.

Regarding meningitis, the incidence was similar to the burden of diseases estimated in 2019. According to this report, the age-standardized rate of meningitis for that year in Andean Latin America was 6.9 cases per 100.000 person-years, which is lower than other regions of Latin America and the Caribbean. The reported mortality was 0.6 cases per 100 000 person-years is higher than in this study.^3^

### Microbiology

This report also aimed to analyze the etiology of meningitis and highlight *S. pneumoniae*, *N. meningitidis,* and *K. pneumoniae* as the main bacterial causes of this infection in patients older than 50 years; however, relevant information was not available. The bacteria associated with the disease are of interest to Colombian public health, and healthcare institutions are mandated to report them to the Colombian National Health Institute.^27^ According to a report of the National Health Institute in 2022, 42.7% of the cases were caused by *S. pneumoniae,* 11.5% by *H. influenzae,* and 9.8% by *N. meningitidis.* The CFR of *S. pneumoniae* meningitis was 18.5% compared with 12.5% for *N. meningitidis* and 9.8% for *H. influenzae.*^27^

### Comorbidity

This study estimated the incidence for meningitis, bacteremia, and pneumonia in a population with different comorbidities in which the incidence of cardiovascular disease, chronic pulmonary disease, dementia, kidney disease, peripheral vascular disease, and hemiplegia/paraplegia were predominant. A similar study in Germany focused on pneumonia reported that the leading comorbidities in patients between 50 and 59 years (incidence >2000 cases per 100 000 person-years) were chronic lung disease, asthma, neuromuscular disorder, lupus, asplenia, HIV, chronic renal failure, malignant neoplasms, solid organ transplantation, and congenital immunodeficiency.^28^ Several studies conducted in the US, Japan, and Spain have demonstrated that some comorbidities increase the risk of meningitis, the main presentation of invasive pneumococcal disease. These conditions include chronic heart disease, chronic lung disease, diabetes mellitus, asplenia, HIV infection, cancer, chronic renal disease, and organ transplantation, with higher incidences than in the healthy population.^29–31^

Although the study did not compare the impact of multiple comorbidities in the incidence due to the unavailability of population at risk for this condition, another study evaluated the increase of incidence based on multimorbidity. Grant et al found that for all-cause pneumonia and invasive pneumonia disease, the incidence was higher in individuals with 2 or more chronic medical conditions compared with healthy population, but similar for those with 1 or 2 medical conditions.^30^

Latin American studies have reported that increasing age was associated with an increase in the incidence of pneumonia, and the probability of death^20^; this coincides with the results of the present study population, in which 1390 cases of hospital pneumonia and 3360 cases of ambulatory pneumonia were reported in people over 70 years. With respect to mortality, the same scenario was observed, with the population over 70 years of age accounting for more than 50% of mortality among the population infected with pneumonia, both inpatient and outpatient.

## COVID

One study using Invasive Respiratory Infection Surveillance analyzed the impact at during early months of the COVID-19 pandemic in the incidence of invasive diseases of bacteria (*S. pneumoniae, H. influenzae*, and *N. meningitidis*) typically transmitted via respiratory droplets. It involved 26 countries in which a sustained reduction in incidence was observed, especially in *S. pneumoniae,* which decreased by 68% at 4 weeks and 82% at 8 weeks.^32^ A US study, examining additional bacterial causes, found that the incidence of *S. pneumoniae*, *H. influenzae*, group A streptococcus, and group B streptococcus in invasive bacterial disease were 58%, 60%, 28%, and 12% lower than previous years, respectively.^33^ Thus, the changes in incidence observed in this study were consistent with other studies that concluded the implementation of containment strategies, the increase of hygiene measures, and interruption of daily activities reduced the transmission of bacteria that have a similar pathway to COVID-19.

### Vaccination

Considering the higher burden on diseases of these infections, some healthcare systems have decided to implement vaccination programs to reduce the incidence, mortality, and cost of managing these events. For example, pneumococcal vaccination in the US has demonstrated a reduction in invasive pneumococcal disease incidence of 57% in adults 19 to 64 years with immunocompromising condition and 68% in adults aged over 65 years.^34^ The strategic Advisory Group of Experts on Immunization from the WHO recommended influenza vaccination for 75% of the population.^35^ In Colombia, adult vaccination programs are relatively weak because the national immunization program focuses on children. According to Andes University, estimated influenza vaccination coverage is nearly 30% in persons aged 60 years or older.^36^ In pneumococcal infection, 19A (21.1%), 3 (12.6%), 4 (9.78%), and 23A (7.6%) serotypes were the most frequent in 2023 according to the Health National Institute of Colombia.^37^

Pneumonia imposes a financial burden on the healthcare system. Vaccination is proposed as a preventive strategy, as it can reduce disease rates and consequently the costs associated to hospitalization and work absenteeism.^38^ The average cost of care for *S. pneumoniae* is Col $12 264 174 ± 14 264 174, due mainly to medications (44%) and hospital stay (35%).^39^ Another clinical event in pneumococcal infection associated with increased cost is MACE, which has a prevalence rate of nearly 23% in invasive pneumococcal disease and 28% to 30% in community-acquired pneumonia.^40^

In Colombia, SALUDATA reported that in 2017, pneumonia represented the fifth leading cause of death in all age groups in Bogotá.^41^ In this study, the mortality trend among adults hospitalized with pneumonia in Colombia was higher between 2015 (24.2%) and 2019 (29.3%), exceeding the mortality rates in 2020 (18%) and 2021 (14.6%), during the COVID-19 pandemic.

In Latin America as of 2024, Argentina, Chile, and Peru have incorporated vaccines for *S. pneumoniae* and influenza virus into their National Immunization Plans for the older adult population. Meanwhile Colombia, Uruguay, and Ecuador fund only vaccines for influenza virus. Brazil does not include these older adult vaccinations. In all North American countries, vaccines for *S. pneumoniae* and influenza virus are covered for the older adult population.^42^ Influenza vaccination coverage across all risk groups is above the regional average in Argentina, Bolivia, Brazil, Chile, Colombia, and Ecuador and below average in Paraguay, Peru, Uruguay, and Venezuela.^42^

### Limitations

This study used a healthcare database including claims databases that depend on ICD codes, consistent with previous studies. However, the presence of a diagnostic code on a medical record does not imply the patient has/had the disease, as the diagnosis code could have been incorrect or included as a rule-out criterion, generating misclassification bias. Unfortunately, it was not possible to validate the diagnosis due to the type of information in the data sources used. Due to the retrospective nature of this study, the data quality determines the results that will be obtained, which may limit generalizability. Third, this study can only demonstrate association and not causation, as it is a retrospective observational study. Fourth, it is not possible to determine the microbial etiology for patients of any infection because of data limitations. Fifth, mortality was considered based on the reported cause of a person’s death at the time, without confirmation that the person died from that disease. Sixth, the results from the pairwise comparison tests for comorbidities should be interpreted with caution as the independence assumption is violated. Finally, the HMO studied represents approximately 10% of Colombia’s population, and the distribution is not homogeneous. It is nonrepresentative of the low-income population, those subsidized by the government, or the rural population. Therefore, the results and conclusion may not be generalizable to the entire population.

## Strengths

This is the first study in the country that researches the total population from one HMO, which allows an estimation of the incidence of these infections. Additionally, it was possible to analyze the behavior in patients with any studied comorbidities considered by the US Centers for Disease Control and Prevention as a risk factor for pneumococcal disease.^16^ Finally, decision makers can use this information to prioritize different interventions in the population to reduce the incidence of these diseases and their mortality.

This study aimed to highlight public health issues with pneumonia, bacteremia, and meningitis, particularly in older people or adults with comorbidities. Addressing these issues requires improving the continuing surveillance by the government to monitor the trend of the diseases and assessing the impact of different strategies to reduce the incidence of these infections. Additionally, evaluating the vaccination strategies that have been implemented in other countries helping to vulnerable people is recommended.

## CONCLUSION

The incidence of meningitis, inpatient pneumonia, outpatient pneumonia, and bacteremia with unknown bacterial cause observed in the HMO suggests a strong correlation with age and comorbidities. This pattern is also present in all-cause mortality. CFR was higher in patients with bacteremia than in those with meningitis and pneumonia; however, these results need to be interpreted cautiously due to limitations in accurately identifying the cause of death. Additional studies are recommended to understand the etiology of these infections at population level as well as their lethality. Although the study has some limitations, including information from a single insurer and reliance on ICD-10 codes, it provides valuable insights on the burden and distribution of these diseases.

### Disclosures

J.M.R., J.A., and J.O. received support from Pfizer for this manuscript. Additionally, J.O. and M.A.F. hold stock, stock options, or other financial or nonfinancial interests from Pfizer.

## Supplementary Material

Online Supplementary Material
