# Supplementary material for: Frequency and Mortality of Adult Meningitis, Pneumonia, or Bacteremia in Colombia from 2015 to 2022: A Retrospective Database Study in a Health Maintenance Organization
Source: J Health Econ Outcomes Res. 2025 Sep 17;12(2):141461. doi: 10.36469/001c.141461 (PMC12448433; doi:10.36469/001c.141461)
Supplement: Online Supplementary Material [file jheor_2025_12_2_141461_300814.pdf]

Journal of Health Economics and Outcomes Research

Online Supplementary Material

Frequency and Mortality of Adult Meningitis, Pneumonia, or Bacteremia in Colombia from 2015 to 2022: A Retrospective Database Study in a Health Maintenance Organization. *JHEOR*. 2025;12(2):??-??. doi:10.36469/jheor.2025.141461

Table S1: Pairwise Comparison Tests of Comorbidity Incidence in Inpatient Pneumonia for Patients Aged Between 51 and 69 Years Using Bonferroni Correction

Table S2: Pairwise Comparison Tests of Comorbidity Incidence in Inpatient Pneumonia for Patients Aged 70 Years or Older Using Bonferroni Correction

Table S3: Pairwise Comparison Tests of Comorbidity Incidence in Outpatient Pneumonia for Patients Aged Between 51 and 69 Years Using Bonferroni Correction

Table S4: Pairwise Comparison Tests of Comorbidity Incidence in Outpatient Pneumonia for Patients Aged 70 Years or Older Using Bonferroni Correction

Table S5: Pairwise Comparison Tests of Comorbidity Incidence in Bacteremia for Patients Aged Between 51 and 69 Years Using Bonferroni Correction

Table S6: Pairwise Comparison Tests of Comorbidity Incidence in Bacteremia for Patients Aged 70 Years or Older Using Bonferroni Correction

Table S7: Beta Coefficients of Negative Binomial Regression With Standard Errors for Meningitis

Table S8: Beta Coefficients of Negative Binomial Regression With Standard Errors for Inpatient Pneumonia

Table S9: Beta Coefficients of Negative Binomial Regression With Standard Errors for Outpatient Pneumonia

Table S10: Beta Coefficients of Negative Binomial Regression With Standard Errors for Bacteremia

This supplementary material has been provided by the authors to give readers additional information about their work.

This is an open-access article distributed under the terms of the Creative Commons Attribution 4.0 International License (CCBY-4.0). View this license's legal deed at http://creativecommons.org/licenses/by/4.0 and legal code at http://creativecommons.org/licenses/by/4.0/legalcode for more information.

Table S11: Incidence of Outpatient Pneumonia by Comorbidities and Age (Cases per 100 000 Persons per Year)

Table S12: Incidence of Inpatient Pneumonia by Comorbidities and Age (Cases per 100 000 Persons per Year)

Table S13: Incidence of Bacteremia by Comorbidities and Age (Cases per 100 000 Persons per Year)

Table S14: Incidence of Meningitis by Comorbidities and Age (Cases per 100 000 Persons per Year)

Table S15: Mortality of Outpatient Pneumonia by Comorbidities and Age (Cases per 100 000 Persons per Year)

Table S16: Mortality of Inpatient Pneumonia by Comorbidities and Age (Cases per 100 000 Persons per Year)

Table S17: Mortality of Meningitis by Comorbidities and Age (Cases per 100 000 Persons per Year)

Table S18: Mortality of Bacteremia by Comorbidities and Age (Cases per 100 000 Persons per Year)

Statistical Differences by Comorbidity

To test statistical differences by comorbidity, pairwise comparisons were developed using a 2-proportion *z*-test and Bonferroni correction. In total, 28 tests were made per disease and age group. Resulting matrices with the corresponding *P* values are displayed below.

Table S1. Pairwise Comparison Tests of Comorbidity Incidence in Inpatient Pneumonia for Patients Aged Between 51 and 69 Years Using Bonferroni Correction

	**Cancer**	**Cardiovascular Disease**	**Chronic Pulmonary Disease**	**Connective Tissue Disease**	**Dementia**	**Diabetes**	**Kidney Disease, Any Stage**
Cardiovascular disease	0.000[c]	1	-	-	-	-	-
Chronic pulmonary disease	0.000[c]	0.044	1	-	-	-	-
Connective tissue disease	0.824	0.000[c]	0.000[c]	1	-	-	-
Dementia	0.000[c]	0.282	0.736	0.000[c]	1	-	-
Diabetes	0.007	0.000[c]	0.000[c]	0.0447	0.000[c]	1	
Kidney disease, any stage	0.000[c]	0.888	0.016	0.000[c]	0.283	0.000[c]	1
Peripheral vascular disease	0.004	0.003[a]	0.000[c]	0.022	0.000[c]	0.000[c]	0.001[b]

[a]$\alpha = 0.1$.
[b]$\alpha = 0.05$.
[c]$\alpha = 0.01$.

,

Table S2. Pairwise Comparison Tests of Comorbidity Incidence in Inpatient Pneumonia for Patients Aged 70 Years or Older Using Bonferroni Correction

	**Cancer**	**Cardiovascular Disease**	**Chronic Pulmonary Disease**	**Connective Tissue Disease**	**Dementia**	**Diabetes**	**Kidney Disease, Any Stage**
Cardiovascular disease	0.000	1	–	–	–	–	–
Chronic pulmonary disease	0.000[c]	0.070	1	–	–	–	–
Connective tissue disease	0.005	0.000[c]	0.000[c]	1	–	–	–
Dementia	0.000[c]	0.000[c]	0.000[c]	0.887	1	–	–
Diabetes	0.776	0.000[b]	0.000[b]	0.002[a]	0.000[b]	1	
Kidney disease, any stage	0.000[b]	0.001[a]	0.001[a]	0.000[b]	0.000[b]	0.000[b]	1
Peripheral vascular disease	0.000[b]	0.000[b]	0.000[b]	0.563	0.548	0.000[b]	0.000[b]

[a]$\alpha = 0.1$.
[b]$\alpha = 0.05$.
[c]$\alpha = 0.01$.

Table S3. Pairwise Comparison Tests of Comorbidity Incidence in Outpatient Pneumonia for Patients Aged Between 51 and 69 Years Using Bonferroni Correction

	**Cancer**	**Cardiovascular Disease**	**Chronic Pulmonary Disease**	**Connective Tissue Disease**	**Dementia**	**Diabetes**	**Kidney Disease, Any Stage**
Cardiovascular disease	0.000[a]	1	–	–	–	–	–
Chronic pulmonary disease	0.000[a]	0.000[a]	1	–	–	–	–
Connective tissue disease	0.000[a]	0.000[a]	0.000[a]	1	–	–	–
Dementia	0.000[a]	0.000[a]	0.000[a]	0.691	1	–	–
Diabetes	0.000[a]	0.000[a]	0.000[a]	0.000[a]	0.000[a]	1	
Kidney disease, any stage	0.000[a]	0.171	0.000[a]	0.000[a]	0.001[a]	0.000[a]	1
Peripheral vascular disease	0.000[a]	0.297	0.000[a]	0.000[a]	0.000[a]	0.000[a]	0.022

Abbreviation: NS, not significant.
[a]$\alpha = 0.01$.

Table S4. Pairwise Comparison Tests of Comorbidity Incidence in Outpatient Pneumonia for Patients Aged 70 Years or Older Using Bonferroni Correction

	Cancer	Cardiovascular Disease	Chronic Pulmonary Disease	Connective Tissue Disease	Dementia	Diabetes	Kidney Disease, Any Stage
Cardiovascular disease	0.000[b]	1	–	–	–	–	–
Chronic pulmonary disease	0.000[b]	0.000[b]	1	–	–	–	–
Connective tissue disease	0.000[b]	0.000[b]	0.000[b]	1	–	–	–
Dementia	0.001[a]	0.000[b]	0.000[b]	0.011	1	–	–
Diabetes	0.001[a]	0.000[b]	0.000[b]	0.000[b]	0.000[b]	1	
Kidney disease, any stage	0.000[b]	0.005	0.000[b]	0.281	0.001[a]	0.000[b]	1
Peripheral vascular disease	0.000[b]	0.616	0.000[b]	0.000[b]	0.000[b]	0.000[b]	0.004

[a]$\alpha = 0.05$.
[b]$\alpha = 0.01$.

Table S5. Pairwise Comparison Tests of Comorbidity Incidence in Bacteremia for Patients Aged Between 51 and 69 Years Using Bonferroni Correction

	Cancer	Cardiovascular Disease	Chronic Pulmonary Disease	Connective Tissue Disease	Dementia	Diabetes	Kidney Disease, Any Stage
Cardiovascular disease	0.039	1	–	–	–	–	–
Chronic pulmonary disease	0.912	0.048	1	–	–	–	–
Connective tissue disease	0.126	0.004	0.112	1	–	–	–
Dementia	0.023	0.633	0.027	0.003[a]	1	–	–
Diabetes	0.000[b]	0.000[b]	0.000[b]	0.436	0.000[b]	1	
Kidney disease, any stage	0.000[b]	0.000[b]	0.000[b]	0.000[b]	0.016	0.000[b]	1
Peripheral vascular disease	0.645	0.076	0.608	0.550	0.041	0.196	0.000[b]

[a]$\alpha = 0.1$.
[b]$\alpha = 0.01$.

Table S6. Pairwise Comparison Tests of Comorbidity Incidence in Bacteremia for Patients Aged 70 Years or Older Using Bonferroni Correction

	Cancer	Cardiovascular Disease	Chronic Pulmonary Disease	Connective Tissue Disease	Dementia	Diabetes	Kidney Disease, Any Stage
Cardiovascular disease	0.000[b]	1	–	–	–	–	–
Chronic pulmonary disease	0.000[b]	0.032	1	–	–	–	–
Connective tissue disease	0.657	0.001[a]	0.040	1	–	–	–
Dementia	0.615	0.000[b]	0.000[b]	0.896	1	–	–
Diabetes	0.922	0.000[b]	0.000[b]	0.679	0.629	1	
Kidney disease, any stage	0.000[b]	0.000[b]	0.000[b]	0.000[b]	0.000[b]	0.000[b]	1
Peripheral vascular disease	0.521	0.045	0.045	0.896	0.759	0.535	0.000[b]

[a]$\alpha = 0.05$.
[b]$\alpha = 0.01$.

Statistical Model

To assess changes in incidences, mortality and CFR of pneumonia (inpatient and outpatient), bacteremia and meningitis by age group and comorbidities negative binomial regression was implemented due to the presence of overdispersion in the data. The number of cases for age group i and comorbidity j are denoted by y_{ij}, which is assumed to follow a negative binomial distribution with mean μ_{ij} following the expression:

$$\log(\mu_{ij}) = \beta_0 + \beta_1 \ age\ group_i + \beta_2 \ comorbidity_j + \log(population_{ij})$$

As shown above, a log link function was used, and an offset term was included to adjust the number of cases by population size, allowing the model to estimate incidence rates while preserving the count nature of the data. The model was fitted independently for each infection and was implemented in R using the glm.nb function from the MASS package.

The assumptions of the model were evaluated. The Durbin-Watson test was performed to assess the independence of observations. The linearity assumption was checked graphically, and variance inflation factors (VIFs) were computed to detect multicollinearity among variables. There were either null or mild violations of the independence assumption, indicating no harm to inference. The residuals behaved well, supporting the linearity assumption. Finally, all VIFs values equaled 1; therefore, no multicollinearity among predictors was found.

The coefficients of the models for the incidences are reported in Tables S1, S2, S3 and S4 for meningitis, inpatient pneumonia, outpatient pneumonia, and bacteremia, respectively. The level of reference in the age groups is 18-50 years in diabetes comorbidities, as is the one with lower incidences.

This information is reported for transparency and completeness but is outside the scope of the study.

Table S7. Beta Coefficients of Negative Binomial Regression With Standard Errors for Meningitis

Age Group	Estimate	IRR	Standard Error	*P* Value
51-60	-0.424	0.655	0.383	.268
61-69	-0.084	0.920	0.346	.808
70+	-0.154	0.857	0.318	.629

Abbreviation: IRR, incidence rate ratio.

Table S8. Beta Coefficients of Negative Binomial Regression With Standard Errors for Inpatient Pneumonia

	Estimate	IRR	Standard Error	*P* Value
Age group				
51-60 y	0.534	1.705	0.097	<.000
61-69 y	0.771	2.162	0.093	<.000
≥70 y	1.508	4.518	0.089	<.000
Comorbidity				
Cancer	0.102	1.108	0.108	.346
Cardiovascular disease	1.043	2.837	0.122	<.000
Chronic pulmonary disease	1.098	2.999	0.104	<.000
Connective tissue disease	0.296	1.345	0.120	.013
Dementia	0.837	2.310	0.131	<.000
Kidney disease at any stage	1.053	2.866	0.112	<.000
Peripheral vascular disease	0.450	1.569	0.132	.001

Abbreviation: IRR, incidence rate ratio.

Table S9. Beta Coefficients of Negative Binomial Regression With Standard Errors for Outpatient Pneumonia

	Estimate	IRR	Standard Error	*P* Value
Age group				
51-60 y	0.252	1.286	0.056	<.000
61-69 y	0.537	1.711	0.054	<.000
≥70 y	0.794	2.212	0.053	<.000
Comorbidity				
Cancer	0.119	1.126	0.068	.079
Cardiovascular disease	0.816	2.260	0.074	<.000
Chronic pulmonary disease	1.134	3.107	0.066	<.000
Connective tissue disease	0.442	1.555	0.070	<.000
Dementia	0.336	1.399	0.082	<.000
Kidney disease, any stage	0.751	2.120	0.070	<.000
Peripheral vascular disease	0.835	2.304	0.077	<.000

Abbreviation: IRR, incidence rate ratio.

Table S10. Beta Coefficients of Negative Binomial Regression With Standard Errors for Bacteremia

	Estimate	IRR	Standard Error	*P* Value
Age group				
51-60 y	0.323	1.381	0.140	.021
61-69 y	0.532	1.703	0.135	[redacted]
≥70 y	1.128	3.091	0.129	<.000
Comorbidity				
Cancer	0.171	1.186	0.159	.284
Cardiovascular disease	0.713	2.039	0.183	<.000
Chronic pulmonary disease	0.199	1.220	0.159	.210
Connective tissue disease	0.155	1.168	0.175	.376
Dementia	0.414	1.513	0.200	.039
Kidney disease, any stage	1.306	3.692	0.161	<.000
Peripheral vascular disease	0.221	1.247	0.196	.260

Abbreviation: IRR, incidence rate ratio.

Table S11. Incidence of Outpatient Pneumonia by Comorbidities and Age (Cases per 100 000 Persons per Year)

Comorbidities, by Age	Incidence Rate	Lower Limit	Upper Limit
Age 18-50 y			
Cancer	2312.11	2175.13	2455.46
Cardiovascular disease	4109.59	3328.78	5018.51
Chronic pulmonary disease	4976.3	4805.15	5151.99
Connective tissue disease	2440.97	2197.6	2703.94
Dementia	3184.08	2177.91	4494.97
Diabetes	1992.65	1859.38	2132.96
Kidney disease, any stage	4514.07	4046.95	5020.32
Peripheral vascular disease	2774.27	2145.62	3529.56
Age 51-60 y			
Cancer	2628.39	2430.34	2838.29
Cardiovascular disease	4415.58	3743.64	5173.32
Chronic pulmonary disease	8102.91	7733.97	8484.89
Connective tissue disease	3389.23	3008.42	3804.89
Dementia	4078.55	3063.93	5321.62
Diabetes	2286.68	2155.62	2423.62
Kidney disease, any stage	5023.35	4481.35	5612.82
Peripheral vascular disease	5112.47	4159.71	6218.14
Age 61-69 y			
Cancer	3221.04	3013.33	3439.3
Cardiovascular disease	7227.07	6469.39	8049.12
Chronic pulmonary disease	10 177.79	9793.15	10 573.68
Connective tissue disease	5575	5060.45	6127.69
Dementia	4383.48	3685.07	5175.74
Diabetes	2822.7	2689.36	2960.93
Kidney disease, any stage	5967.59	5467.04	6501.66
Peripheral vascular disease	7551.67	6516.02	8705.15
Age ≥70 y			
Cancer	4289.07	4084.22	4501.53
Cardiovascular disease	9866.77	9172.26	10 599.93
Chronic pulmonary disease	11 830.12	11 511.44	12 155.39
Connective tissue disease	6511.51	5959.63	7100.75
Dementia	4926.71	4686.22	5176.34
Diabetes	4086.54	3946.08	4230.72
Kidney disease, any stage	7985.63	7599.09	8386.73
Peripheral vascular disease	10 939.3	9976.54	11 969.89

Table S12. Incidence of Inpatient Pneumonia by Comorbidities and Age (Cases per 100 000 Persons per Year)

Comorbidities, by Age	Incidence Rate	Lower Limit	Upper Limit
Age 18-50 y			
Cancer	232.95	190.91	281.5
Cardiovascular disease	1027.4	658.27	1528.69
Chronic pulmonary disease	603.33	544.71	666.54
Connective tissue disease	332.56	246.83	438.44
Dementia	995.02	477.15	1829.89
Diabetes	232.88	188.85	284.09
Kidney disease, any stage	955.92	747.95	1203.82
Peripheral vascular disease	252.21	92.56	548.95
Age 51-60 y			
Cancer	448.16	368.67	539.7
Cardiovascular disease	1356.42	996.65	1803.75
Chronic pulmonary disease	1355.7	1207.34	1517.27
Connective tissue disease	472.37	337.47	643.23
Dementia	1510.57	922.7	2332.96
Diabetes	368.36	316.93	425.77
Kidney disease, any stage	1239.74	978.38	1549.46
Peripheral vascular disease	664.62	353.88	1136.52
Age 61-69 y			
Cancer	601.69	513.9	700.19
Cardiovascular disease	1222.71	923.62	1587.79
Chronic pulmonary disease	1770.22	1611.96	1939.82
Connective tissue disease	622.33	458.85	825.11
Dementia	1513.72	1116.1	2006.97
Diabetes	483.6	429.36	542.8
Kidney disease, any stage	1346.42	1114.47	1612.41
Peripheral vascular disease	1788.55	1304.58	2393.22
Age ≥70 y			
Cancer	1354.03	1240.03	1475.69
Cardiovascular disease	2862.42	2494.21	3269.67
Chronic pulmonary disease	3259.34	3093.1	3432.2
Connective tissue disease	1767.77	1486.12	2087.28
Dementia	1791.53	1647.7	1944.55
Diabetes	1333.62	1253.91	1417.08
Kidney disease, any stage	2745.06	2520.41	2984.36
Peripheral vascular disease	3831.06	3270.43	4460.23

Table S13. Incidence of Bacteremia by Comorbidities and Age (Cases per 100 000 Persons per Year)

Comorbidities, by Age	Incidence Rate	Lower Limit	Upper Limit
Age 18-50 y			
Cancer	174.17	138.11	216.77
Cardiovascular disease	470.89	235.07	842.55
Chronic pulmonary disease	101.33	78.21	129.16
Connective tissue disease	246.09	173.27	339.21
Dementia	298.51	61.56	872.37
Diabetes	194.46	154.43	241.7
Kidney disease, any stage	1088.69	865.87	1351.35
Peripheral vascular disease	336.28	145.18	662.6
Age 51-60 y			
Cancer	375.48	303.06	459.99
Cardiovascular disease	490.62	285.8	785.53
Chronic pulmonary disease	362.42	287.81	450.45
Connective tissue disease	259.8	162.82	393.34
Dementia	453.17	166.31	986.37
Diabetes	199.28	161.96	242.62
Kidney disease, any stage	917.73	695.08	1189.02
Peripheral vascular disease	409	176.58	805.89
Age 61-69 y			
Cancer	381.91	312.68	461.91
Cardiovascular disease	567.69	370.83	831.79
Chronic pulmonary disease	401.1	327.72	485.99
Connective tissue disease	337.09	220.2	493.92
Dementia	662.25	409.94	1012.32
Diabetes	315.69	272.17	364.18
Kidney disease, any stage	1038.34	836.01	1274.85
Peripheral vascular disease	675.68	393.61	1081.82
Age ≥70 y			
Cancer	654.84	576.33	741.06
Cardiovascular disease	1200.37	966.46	1473.79
Chronic pulmonary disease	937.71	849.55	1032.54
Connective tissue disease	699.48	526.94	910.47
Dementia	685.92	598.08	783.03
Diabetes	659.78	604.07	719.24
Kidney disease, any stage	1442.4	1280.88	1618.66
Peripheral vascular disease	1453.96	1117.26	1860.24

Table S14. Incidence of Meningitis by Comorbidities and Age (Cases per 100 000 Persons per Year)

**Comorbidities, by Age**	**Incidence Rate**	**Lower Limit**	**Upper Limit**
Age 18-50 y			
Cancer	28.3	15.07	48.4
Cardiovascular disease	42.81	1.08	238.51
Chronic pulmonary disease	12.47	5.38	24.57
Connective tissue disease	19.95	4.11	58.31
Dementia	99.5	2.52	554.39
Diabetes	7.2	1.49	21.05
Kidney disease, any stage	66.38	21.55	154.92
Peripheral vascular disease	0	0	155.06
Age 51-60 y			
Cancer	8.07	0.98	29.17
Cardiovascular disease	0	0	106.46
Chronic pulmonary disease	4.47	0.11	24.93
Connective tissue disease	35.43	7.31	103.53
Dementia	75.53	1.91	420.82
Diabetes	10.06	3.27	23.49
Kidney disease, any stage	32.2	3.9	116.32
Peripheral vascular disease	51.12	1.29	284.85
Age 61-69 y			
Cancer	14.41	3.93	36.9
Cardiovascular disease	21.83	0.55	121.65
Chronic pulmonary disease	7.71	0.93	27.86
Connective tissue disease	25.93	3.14	93.67
Dementia	126.14	34.37	322.98
Diabetes	6.72	1.83	17.2
Kidney disease, any stage	34.23	7.06	100.04
Peripheral vascular disease	79.49	9.63	287.15
Age ≥70 y			
Cancer	18.26	7.34	37.63
Cardiovascular disease	39.57	8.16	115.65
Chronic pulmonary disease	20.39	9.32	38.7
Connective tissue disease	38.15	7.87	111.5
Dementia	12.53	3.41	32.08
Diabetes	11.51	5.26	21.85
Kidney disease, any stage	19.96	5.44	51.12
Peripheral vascular disease	23.08	0.58	128.59

Table S15. Mortality of Outpatient Pneumonia by Comorbidities and Age (Cases per 100 000 Persons per Year)

Comorbidities, by Age	Incidence Rate	Lower Limit	Upper Limit
Age 18-50 y			
Cancer	28.3	15.07	48.4
Cardiovascular disease	42.81	1.08	238.51
Chronic pulmonary disease	12.47	5.38	24.57
Connective tissue disease	19.95	4.11	58.31
Dementia	99.5	2.52	554.39
Diabetes	7.2	1.49	21.05
Kidney disease, any stage	66.38	21.55	154.92
Peripheral vascular disease	0	0	155.06
Age 51-60 y			
Cancer	8.07	0.98	29.17
Cardiovascular disease	0	0	106.46
Chronic pulmonary disease	4.47	0.11	24.93
Connective tissue disease	35.43	7.31	103.53
Dementia	75.53	1.91	420.82
Diabetes	10.06	3.27	23.49
Kidney disease, any stage	32.2	3.9	116.32
Peripheral vascular disease	51.12	1.29	284.85
Age 61-69 y			
Cancer	14.41	3.93	36.9
Cardiovascular disease	21.83	0.55	121.65
Chronic pulmonary disease	7.71	0.93	27.86
Connective tissue disease	25.93	3.14	93.67
Dementia	126.14	34.37	322.98
Diabetes	6.72	1.83	17.2
Kidney disease, any stage	34.23	7.06	100.04
Peripheral vascular disease	79.49	9.63	287.15
Age ≥70 y			
Cancer	18.26	7.34	37.63
Cardiovascular disease	39.57	8.16	115.65
Chronic pulmonary disease	20.39	9.32	38.7
Connective tissue disease	38.15	7.87	111.5
Dementia	12.53	3.41	32.08
Diabetes	11.51	5.26	21.85
Kidney disease, any stage	19.96	5.44	51.12
Peripheral vascular disease	23.08	0.58	128.59

Table S16. Mortality of Inpatient Pneumonia by Comorbidities and Age (Cases per 100 000 Persons per Year)

Comorbidities, by Age	Incidence Rate	Lower Limit	Upper Limit
Age 18-50 y			
Cancer	67.49	45.86	95.8
Cardiovascular disease	342.47	147.85	674.79
Chronic pulmonary disease	54.56	38.01	75.89
Connective tissue disease	26.6	7.25	68.12
Dementia	398.01	108.44	1019.06
Diabetes	24.01	11.51	44.15
Kidney disease, any stage	132.77	63.67	244.16
Peripheral vascular disease	126.1	26.01	368.53
Age 51-60 y			
Cancer	161.5	115.38	219.92
Cardiovascular disease	173.16	63.55	376.9
Chronic pulmonary disease	174.5	124.08	238.54
Connective tissue disease	94.47	40.79	186.15
Dementia	604.23	260.86	1190.57
Diabetes	74.48	52.44	102.66
Kidney disease, any stage	209.31	111.45	357.92
Peripheral vascular disease	102.25	12.38	369.36
Age 61-69 y			
Cancer	241.4	187.08	306.57
Cardiovascular disease	371.18	216.23	594.29
Chronic pulmonary disease	381.81	310.32	464.84
Connective tissue disease	103.72	44.78	204.37
Dementia	756.86	484.93	1126.15
Diabetes	136.01	108.01	169.05
Kidney disease, any stage	342.31	230.95	488.67
Peripheral vascular disease	596.18	333.68	983.32
Age ≥70 y			
Cancer	600.05	525	682.82
Cardiovascular disease	1516.95	1252.4	1820.87
Chronic pulmonary disease	1263.87	1161.17	1373.23
Connective tissue disease	674.04	504.9	881.66
Dementia	1058.63	948.77	1177.73
Diabetes	539.59	489.33	593.61
Kidney disease, any stage	1152.92	1009.03	1311.58
Peripheral vascular disease	1707.82	1341.01	2144.02

Table S17. Mortality of Meningitis by Comorbidities and Age (Cases per 100 000 Persons per Year)

Comorbidities, by Age	Incidence Rate	Lower Limit	Upper Limit
Age 18-50 y			
Cancer	6.53	1.35	19.09
Cardiovascular disease	0	0	157.91
Chronic pulmonary disease	1.56	0.04	8.69
Connective tissue disease	0	0	24.54
Dementia	0	0	367.05
Diabetes	0	0	8.86
Kidney disease, any stage	26.55	3.22	95.92
Peripheral vascular disease	0	0	155.06
Age 51-60 y			
Cancer	0	0	14.89
Cardiovascular disease	0	0	106.46
Chronic pulmonary disease	4.47	0.11	24.93
Connective tissue disease	11.81	0.3	65.8
Dementia	0	0	278.62
Diabetes	6.04	1.25	17.65
Kidney disease, any stage	16.1	0.41	89.71
Peripheral vascular disease	0	0	188.59
Age 61-69 y			
Cancer	0	0	13.29
Cardiovascular disease	0	0	80.54
Chronic pulmonary disease	0	0	14.23
Connective tissue disease	12.97	0.33	72.24
Dementia	31.54	0.8	175.71
Diabetes	0	0	6.19
Kidney disease, any stage	11.41	0.29	63.57
Peripheral vascular disease	39.75	1.01	221.45
Age ≥70 y			
Cancer	5.22	0.63	18.85
Cardiovascular disease	26.38	3.19	95.3
Chronic pulmonary disease	13.59	4.99	29.58
Connective tissue disease	25.44	3.08	91.88
Dementia	3.13	0.08	17.45
Diabetes	6.39	2.08	14.92
Kidney disease, any stage	0	0	18.41
Peripheral vascular disease	23.08	0.58	128.59

Table S18. Mortality of Bacteremia by Comorbidities and Age (Cases per 100 000 Persons per Year)

Comorbidities, by Age	Incidence Rate	Lower Limit	Upper Limit
Age 18-50 y			
Cancer	60.96	40.51	88.1
Cardiovascular disease	299.66	120.48	617.41
Chronic pulmonary disease	23.38	13.09	38.57
Connective tissue disease	26.6	7.25	68.12
Dementia	99.5	2.52	554.39
Diabetes	28.81	14.89	50.32
Kidney disease, any stage	318.64	204.16	474.11
Peripheral vascular disease	42.03	1.06	234.2
Age 51-60 y			
Cancer	189.76	139.43	252.34
Cardiovascular disease	86.58	17.85	253.02
Chronic pulmonary disease	147.65	101.64	207.36
Connective tissue disease	59.05	19.17	137.79
Dementia	302.11	82.32	773.53
Diabetes	40.26	24.59	62.18
Kidney disease, any stage	209.31	111.45	357.92
Peripheral vascular disease	0	0	188.59
Age 61-69 y			
Cancer	169.34	124.42	225.18
Cardiovascular disease	283.84	151.13	485.38
Chronic pulmonary disease	107.99	71.76	156.07
Connective tissue disease	116.69	53.36	221.51
Dementia	536.11	312.3	858.36
Diabetes	105.79	81.29	135.35
Kidney disease, any stage	319.49	212.3	461.75
Peripheral vascular disease	278.22	111.86	573.24
Age ≥70 y			
Cancer	352.2	295.3	416.88
Cardiovascular disease	672.73	500.89	884.52
Chronic pulmonary disease	437.15	377.65	503.36
Connective tissue disease	368.82	247	529.68
Dementia	385.24	320.17	459.65
Diabetes	301.76	264.49	342.82
Kidney disease, any stage	698.74	587.79	824.54
Peripheral vascular disease	784.68	543.41	1096.51
